# In Response

**DOI:** 10.1213/ANE.0000000000007537

**Published:** 2025-04-17

**Authors:** Kariem El-Boghdadly, Jaideep J. Pandit

**Affiliations:** Department of Anaesthesia, Guy’s and St Thomas’ NHS Foundation Trust, London, UK; University of Oxford, Oxford, UK, Nuffield Department of Clinical Neurosciences, University of Oxford, Oxford, UK, jaideep.pandit@sjc.ox.ac.uk

We thank Ellefsen and Kristensen^1^ for their thoughtful comments and recognize their original contribution to the technique. We confine our response to what they had to say about our editorial.^2^ They claim that their method of placing the tracheal tube 10 to 14 cm from the teeth avoids our concern that the tube tip could migrate into the esophagus.

However, we stress that tube tip in pharynx (TTIP) is fundamentally a blind technique; moreover, one often conducted in the heat of the moment of a cannot-intubate cannot-ventilate scenario. The video evidence they refer to was fibreoptic examination post hoc—after blind tube insertion—and not used as a proactive guide to tube placement. Just because, on average, the tube tip with TTIP may lie away from the esophagus does not exclude the possibility that on occasion, catastrophe may result from TTIP migration into the esophagus, if the specific distance of 10 to 14 cm cannot be maintained.

Whilst transient ventilation may be possible, as suggested by Markham et al.,^3^ this technique carries significant risks that, in the majority of settings, are not justifiable if alternative equipment, such as supraglottic airways or fibreoptic intubation is available. We therefore believe that TTIP should not routinely be advocated in resource-rich environments such as hospitals.


**Kariem El-Boghdadly, FRCA** *Department of Anaesthesia* *Guy’s and St Thomas’ NHS Foundation Trust* *London, UK*
**Jaideep J. Pandit, MA, BMBCH, DPHIL, FRCA, DM** *University of Oxford* *Oxford, UK
Nuffield Department of Clinical Neurosciences,* *University of Oxford* *Oxford, UK* *jaideep.pandit@sjc.ox.ac.uk*

